# Publisher Correction: Interactions of Co, Cu, and non-metal phthalocyanines with external structures of SARS-CoV-2 using docking and molecular dynamics

**DOI:** 10.1038/s41598-022-08312-y

**Published:** 2022-03-14

**Authors:** Wilson Luna Machado Alencar, Tiago da Silva Arouche, Abel Ferreira Gomes Neto, Teodorico de Castro Ramalho, Raul Nunes de Carvalho Júnior, Antonio Maia de Jesus Chaves Neto

**Affiliations:** 1 Laboratory of Preparation and Computation of Nanomaterials (LPCN), Federal University of Pará, C. P. 479, Belem, PA 66075‑110 Brazil; 2 Pos‑Graduation Program in Engineering of Natural Resources of the Amazon, ITEC, Federal University of Pará, C. P. 2626, Belém, PA 66050‑540 Brazil; 3 Pos‑Graduation Program in Chemical Engineering, ITEC, Federal University of Pará, C. P. 479, Belém, PA 66075‑900 Brazil; 4 National Professional Master’s in Physics Teaching, Federal University of Pará, C. P. 479, Belém, PA 66075‑110 Brazil; 5 Federal Institute of Pará (IFPA), C. P. BR 316, Km 61, Castanhal, PA 68740‑970 Brazil; 6 Chemistry Department, Federal University of Lavras (UFLA), C. P. 3037, Lavras, MG 37200‑000 Brazil

Correction to: *Scientific Reports* 10.1038/s41598-022-07396-w, published online 28 February 2022

The Acknowledgements section in the original version of this Article was incomplete.

“The authors are grateful for the support of the Coordination for the Improvement of Higher Education Personnel (CAPES), of the National Council for Scientific and Technological Development (CNPq) of the Federal University of Pará (UFPA).”

now reads:

“The authors are grateful for the support of the PROPESP/UFPA (PAPQ), Coordination for the Improvement of Higher Education Personnel (CAPES), and the National Council for Scientific and Technological Development (CNPq).”

The original Article has been corrected.

